# Four-wheel-drive immune protection

**DOI:** 10.1038/s41392-022-01180-y

**Published:** 2022-09-27

**Authors:** Fabian Heinrich, Susanne Krasemann

**Affiliations:** 1 Institute of Legal Medicine, University Medical Center Hamburg Eppendorf, 20251 Hamburg, Germany; 2 Institute of Neuropathology, University Medical Center Hamburg Eppendorf, 20251 Hamburg, Germany

**Keywords:** Infectious diseases, Outcomes research, Vaccines, Vaccines, Adaptive immunity

**Fig. 1:**
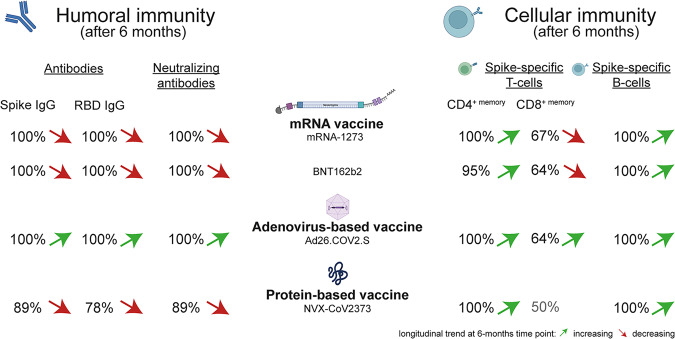
Humoral and cellular immune responses after mRNA- (mRNA-1273, BNT162b2), adenovirus- (Ad26.COV2.S), and recombinant protein-based (NVX-CoV2373) vaccination are illustrated. Responder rates and the longitudinal trend at a 6-month time point are depicted as observed by Zhang et al. and are consistent with the relatively high degree of protection maintained against infection and severe disease after vaccination. Of note, response rates in the humoral and cellular compartment may have peaked before the 6-month time point. mRNA vaccines were consistently observed as the most immunogenic, with immune response levels higher than or equal to the other vaccines. In contrast, Ad26.COV2.S vaccination elicited a generally lower but relatively stable immune response. While NVX-CoV2373-induced neutralizing antibody titers and memory CD4^+^ T cell proportions were comparable to the mRNA vaccines, the induction of spike-specific CD8^+^ T cells was low in NVX-CoV2373 and needs further evaluation. This figure was created using BioRender. RBD receptor-binding domain

In a recent study published in *Cell*, Zhang and colleagues provide the first longitudinal study comparing four different COVID-19 vaccines head-to-head for binding and neutralizing antibodies, spike-specific CD4^+^ or CD8^+^ T cells, and spike- and receptor-binding domain (RBD)-specific memory B cells (Fig. 1).^1^

Evaluating the impact of vaccination on the course of the COVID-19 pandemic is challenging. However, recent calculations estimate that COVID-19 vaccines have saved millions of lives by evoking adaptive immune protection.^2^ This has been mainly achieved by launching two novel vaccine platforms during the pandemic: mRNA- and vector-based vaccines. While high vaccine efficacy (VE) was shown for mRNA-based vaccines, immune correlates mediating protection against SARS-CoV-2 infection, and severe disease remains elusive.

Although serological studies were provided before,^1^ the new study stands out in several aspects: It is the first that used a side-by-side comparison of four different vaccines—among them vaccines from three different vaccine platforms: two mRNA-based vaccines (Moderna mRNA-1273 and Pfizer/BioNTech BNT162b2), a viral vector-based (Janssen/J&J Ad26.COV2.S), and a recombinant spike-protein-based vaccine (Novavax NVX-CoV2373). The authors used comparable workflow and assays for sample preparation and analysis, e.g., a batch preparation of spike-protein-derived peptides, thus, avoiding cross-laboratory differences. Moreover, the authors simultaneously assessed multiple parameters from the same study participants, including an in-depth cellular subset analysis. This is the first longitudinal study that determined the kinetics of vaccine-specific immune memory.

All four vaccine groups were similar in age, gender, and race/ ethnicity distribution, with 30 participants each (12 for NVX-CoV2373). Five different time points were chosen for analyses: pre-vaccination, around days 15, 45, 105, and 185. For NVX-CoV2373, only two time points—days 120 and 185—were assessed. In addition, the immune memory elicited by the vaccines after six months was compared to those of natural infection. Of note, all four vaccines performed equal or superior to natural infection for all measured anti-spike humoral and cellular immune response parameters, which is in line with current results showing that the breadth of the antibody response is much broader after vaccination.^3^

Although antibodies have been established as a correlate of protection, contributions from B and T cells have been suggested. Accordingly, the authors measured antibody titers against spike or RBD and titers of pseudovirus neutralization. Noteworthy, antibody responses were detected in 100% of vaccinated individuals within the time investigated. However, mRNA-based vaccines induced significantly higher titers than vector-based vaccine. Antibody titers changed over time in different patterns: While those for mRNA vaccines dropped, those for Ad26.COV2.S plateaued but was still lower than that of the other three vaccines.

The authors provide a detailed characterization of the spike-specific CD4^+^ T cell responses utilizing flow-cytometry panels that allowed the determination of activation-induced marker (AIM) profiles, intracellular staining of cytokines (ICS), granzyme-B, or intracellular iCD40L. Of note, spike-specific AIM^+^ CD4^+^ T cell responses were detected in 100% of individuals from all four vaccine groups. However, the memory CD4^+^ T cell response after mRNA-1273 vaccination was significantly higher than those elicited by the other vaccines or infection after 6 months. Although mRNA-based vaccines and NVX-CoV2373 induced stably maintained multifunctional CD4^+^ T cells, mRNA-1273 had considerably higher peak responses in all measured ICS CD4^+^ T cell subsets. In contrast, Ad26.COV2.S vaccinees showed lower numbers of responders, a significantly lower percentage of these subpopulations, and lower CD4^+^ cytotoxic lymphocyte percentages. In addition to differences in their mode of action, application characteristics can also be responsible for such variations, e.g., single dose application in Ad26.COV2.S. Heterologous booster application could be a potential strategy here since they have been shown to elicit immune responses that combine specific effects of various vaccines.^4^

Zhang and colleagues also determined the kinetics of spike-specific CD8^+^ cells, again focusing on AIM and ICS profiles. In contrast to CD4^+^ cells, only a maximum number of 83% and 87% responders, respectively, could be detected for mRNA-1273 and an even lower rate for the other three vaccines (BNT162b2 > Ad26.COV2.S > NVX-CoV2373). Overall, memory CD8^+^ T cell frequencies and response rates were similar between mRNA-1273, BNT162b2, and Ad26.COV2.S immunizations. However, multifunctional spike-specific memory CD8^+^ cells were more common in mRNA-1273 recipients. There were only minimal multifunctional cells in NVX-CoV2373 vaccinees, which was expected for a protein-based vaccine.^1^ Of note, the study showed that individual T-cell kinetics fluctuate much more than antibody responses.

Finally, the authors characterized spike- and RBD-specific memory B cells, detected in 100% of vaccinees after 6 months. The relative amount of activated memory B cells was significantly higher for the two mRNA-based vaccines. Memory B cells did not show the same kinetics as antibody responses. Of note, while antibody titers waned over time, the frequency of spike-specific memory B cells increased and, comparable to T cell compartments, remained stable. Of note, spike-specific CXCR3^+^ memory B cells were significantly higher, specifically in Ad26.COV2.S vaccinees at day 105 and after 6 months, similar to infection. CXCR3^+^ memory B cells have been implicated as important modulators of mucosal immunity.^5^ However, their role in mucosal recruitment in the context of the current pandemic needs to be determined.

Since ancient SARS-CoV-2 spike-derived peptides were used in this study, data might substantially differ for novel virus variants. It would be interesting to perform such a thorough longitudinal study in other cohorts, such as children or older people. Nevertheless, this study will stay unique since naive study participants might be impossible to recruit.

In conclusion, Zhang and colleagues underline the high immunogenicity of mRNA vaccines compared to other vaccine platforms by describing more reactive immune responses within the adaptive immune system. More importantly, the novel data will provide a basis to assess and guide future approaches to adapt and improve existing vaccine platforms.
